# Ultrasound-assisted brace casting for adolescent idiopathic scoliosis

**DOI:** 10.1186/1748-7161-10-S1-O38

**Published:** 2015-01-19

**Authors:** Edmond Lou, Amanda Chan, Andreas Donauer, Melissa Tilburn, Doug Hill

**Affiliations:** 1 University of Alberta, Alberta, Canada; 2 Alberta Health Services, Alberta, Canada

## Objectives

There is a short period during rapid growth when a scoliosis brace is most effective. In standard practice, the in-brace correction is not measured during brace casting. The first assessment of the in-brace correction in the follow-up clinic may trigger brace adjustment for improved spinal alignment resulting in delay of effective treatment and increased number of radiographs. The objective was to investigate if real-time ultrasound (US) can aid orthotists in determining the pad pressure level and location resulting in optimal in-brace correction of the spine.

## Materials and methods

Twenty six subjects participated with 9 candidates (2M, 7F) in the intervention group and 17 (2M, 15F) in the control group. All participants were diagnosed with AIS and prescribed either a full time TLSO or a night time Providence brace. A medical ultrasound (US) system, a custom pressure adjustment system and custom software were used.

For the intervention group, each subject first underwent a baseline standing US scan. The orthotist then used a custom standing Providence brace design system to apply pressures against the patient's torso. The applied pad pressures were recorded using our pressure system. The first US assessment scan was acquired. A real-time US spinal image with the coronal curvature measurement was displayed. A comparison between the baseline and the assessment scan was performed. The orthotist then decided if an adjustment was needed in terms of altering the pad locations and magnitudes. Another US scan was taken if a second configuration was required. The orthotist used the best simulated in-brace correction configuration to cast the brace.

## Results

The orthotist tried a second configuration in 7 out of 9 cases (78%). Among these 7 cases, 5 showed better stimulated in-brace corrections which was used to cast the brace. Ultrasound resulted in immediate improved in-brace correction for 5 out of 9 cases (56%). In the control group, 8 of 17 (47%) subjects required a total of 16 additional brace adjustments requiring a total of 33 in-brace radiographs. Some subjects required multiple adjustments. Three intervention subjects have returned to the follow-up in-brace clinics and the other 6 are scheduled in the next 2 months. One subject required a minor adjustment. The total number of in-brace x-rays was 4.

## Conclusions

The use of the ultrasound system provided a radiation-free method to determine the optimum pressure level and location to obtain the best stimulated in-brace correction during brace casting.

